# Plant and Soil Development Cooperatively Shaped the Composition of the *phoD*-Harboring Bacterial Community along the Primary Succession in the Hailuogou Glacier Chronosequence

**DOI:** 10.1128/mSystems.00475-20

**Published:** 2020-07-28

**Authors:** Yan Bai, Quanju Xiang, Ke Zhao, Xiumei Yu, Qiang Chen, Menggen Ma, Hao Jiang, Xiaoping Zhang, Petri Penttinen, Yunfu Gu

**Affiliations:** a Department of Microbiology, College of Resources, Sichuan Agricultural University, Chengdu, Sichuan, China; b Institute of Mountain Hazards and Environment, Chinese Academy of Sciences, Chengdu, Sichuan, China; Institute of Soil Science, Chinese Academy of Sciences

**Keywords:** primary succession, soil development, *phoD* community, distance decay

## Abstract

Microbes that produce phosphatases play an important role in the cycling of phosphorus (P), a key nutrient in soil development. We studied the development, compositional turnover, and environmental drivers of microbial communities carrying the phosphatase-encoding *phoD* gene (here called *phoD* communities) in the course of primary succession in the Hailuogou glacier chronosequence. We selected the pioneer species Populus purdomii Rehder as a model plant to study the communities in rhizosphere and bulk soils along the chronosequence. The bulk and rhizosphere soils hosted distinct *phoD* communities. Changes in the taxa *Pseudomonas* and *Pleomorphomonas* in the rhizosphere and *Bradyrhizobium, Cupriavidus*, and *Pleomorphomonas* in the bulk soil were associated with soil development. The plant development and soil property changes along the chronosequence were accompanied with changes in the *phoD* communities. Soil pH, soil organic carbon, and total nitrogen contents that are directly related to the plant development and litter input differences along the chronosequence were the main factors related to changes in community compositions. The community similarity decreased along the chronosequence, and the distance decay rate was higher in the bulk soil than in the rhizosphere. In summary, both in the rhizosphere and in bulk soils the *phoD* community succession was shaped by plant and soil development-related factors along the primary succession in the Hailuogou glacier chronosequence.

**IMPORTANCE** Phosphorus was the key limiting nutrient for soil development during primary succession that occurred in alpine and high-latitude ecosystems with cold and humid climates. The interactions of functional microbiota involved in phosphorus cycling in the rhizosphere under different soil developmental stages along primary succession are still rarely examined. We selected the pioneer species *Populus purdomii* as a model plant to study the *phoD*-harboring bacterial communities in rhizosphere and bulk soils along a mountain glacier chronosequence. Our results showed that the bulk soils and rhizosphere host distinct *phoD* communities and diversity that differentially varied along the chronosequence, describing in detail the development and compositional turnover of the *phoD* community in the course of primary succession and determining the main environmental factors driving the development.

## Abstract (precis)

Phosphorus was the key limiting nutrient for soil development during primary succession that occurred in alpine and high-latitude ecosystems with cold and humid climates. The interactions of functional microbiota involved in phosphorus cycling in the rhizosphere under different soil developmental stages along primary succession are still rarely examined. We selected the pioneer species *Populus purdomii* as a model plant to study the *phoD*-harboring bacterial communities in rhizosphere and bulk soils along a mountain glacier chronosequence. Our results showed that the bulk soils and rhizosphere host distinct *phoD* communities and diversity that differentially varied along the chronosequence, describing in detail the development and compositional turnover of the *phoD* community in the course of primary succession and determining the main environmental factors driving the development.

## INTRODUCTION

Primary succession is the process in which a region with little to no preexisting life, for example, after glacier retreat or after volcanic eruptions, is colonized by pioneer species and pioneer communities develop (1, 2). Microbes are usually the first colonizers and keystone players in a primary succession, and the dynamic and complex change in microbial community is closely related to the soil development, changes in soil properties, and plant community succession, but the extent to which these factors contribute to microbial communities has not yet been established (3, 4). Most of the studies characterizing the variation and driving factors of microbial communities in a primary succession have historically focused on the species composition of the communities (5, 6). It remains unclear whether similar trends occur in the functional microbial community and how the functional community network structure is shaped during the primary succession. Thus, there is a need to decipher the structure of functional microbial communities along primary succession.

As one of the essential nutrients, phosphorus (P) plays a key role in plant growth and soil development (7). The long-term changes in soil P have important ecological consequences (8). In Puca Glacier, Peru, plant and microbial primary producers were limited by P, not nitrogen, in the early-stage primary succession after glacial retreat (9). Microorganisms can solubilize inorganic P and hydrolyze organic P to orthophosphates through the production of phosphatases (10). When P is scarce, microorganisms upregulate genes in the phosphate (Pho) regulon that includes genes encoding phosphatases and phosphate transporters for mobilizing soil P (11). Phosphatases can potentially hydrolyze up to 90% of the total organic P in soil (10). This underlines the importance of effective Pho regulon control for microorganisms to use alternative P sources during phosphate limitation. In the Pho regulon, *phoD* encodes a monomeric enzyme able to hydrolyze both phosphomonoesters and phosphodiesters (10). The *phoD* gene has been widely found in aquatic and soil environments (10, 12). Vegetation and pH were the key drivers of the *phoD*-harboring community (here called *phoD* community) structure in different soil types (10, 13). To our knowledge, the composition and drivers of the *phoD*-harboring community during primary succession have not been studied.

The Hailuogou glacier chronosequence, located on the southeastern edge of the Tibetan Plateau (14), provides an ideal platform for exploring the relationships between soil development and the succession of microbial and plant communities. The relatively mild and humid climate in the area results in a rapid colonization of moraine by soil microorganisms and plants, accumulation of organic material, and relatively rapid soil development. Following glacier retreat, an approximately 2-km-long belt covering a complete primary succession series from bare land to a lush forest stage has developed. Pedogenesis (7, 15), plant succession (16), transformation of soil organic phosphorus (17), and bacterial and fungal communities in the area (5) have been studied, yet the phosphorus-associated microbial community has not received attention.

In this study, we selected Populus purdomii Rehder as a model plant to study the *phoD* communities because this species was present all along the Hailuogou glacier chronosequence. Our aim was to describe in detail the development and compositional turnover of the *phoD* community in the course of primary succession and to determine the main environmental factors driving the development. We hypothesized that the *phoD* community compositions in the rhizosphere and bulk soils are different, that the role of plants in recruiting the rhizosphere microorganisms is stronger in the early than in the later stages, and that edaphic properties shape the *phoD* community compositions in mature soils.

## RESULTS

### Changes in the soil properties and vegetation along the chronosequence.

*P. purdomii* trees were present at every primary successional stage (see Table S1 in the supplemental material) and therefore selected as the model plant in this study. Based on the soil physicochemical parameters (Table S1), the sampling sites along the Hailuogou glacier chronosequence were assigned into three different developmental stage groups: barren (4- and 22-year-old soils at the first phases of development), developing (40- and 54-year-old soils at the intermediate phase of development), and mature (62-, 89-, and 129-year-old mature soil) (permutational multivariate analysis of variance [PERMANOVA], *F* = 34.04; *P* = 0) (Fig. S2; Table S1). Soil organic carbon (SOC), total nitrogen (TN), and available N (AN) contents and cation exchange capacity (CEC) increased along the chronosequence, whereas soil pH and soil density decreased (Table S1). Total phosphorus (TP) and available P (AP) contents first increased and then decreased along the chronosequence. The AP-to-TP ratio was lowest in the 22-year-old soil and highest in the 89-year-old soil. The TN-to-TP ratio was lowest in the 22-year-old soil and highest in the 129-year-old soil. The concentrations of different soil P fractions were generally low in the barren soil and high in the mature soil (Table S1). The annual litter biomass became higher along the development stages (Table S1).

10.1128/mSystems.00475-20.2 FIG S2 Grouping of the seven Hailuogou glacier chronosequence sampling sites into barren, developing, and mature developmental stages based on the canonical analysis of principal coordinates of the soil properties. Download FIG S2, PDF file, 0.1 MB. Copyright © 2020 Bai et al. 2020 Bai et al. This content is distributed under the terms of the Creative Commons Attribution 4.0 International license.

10.1128/mSystems.00475-20.6 TABLE S1 Basic information on the Hailuogou chronosequence and sampling sites, the characteristics of *Populus purdomii*, soil properties and concentration of soil phosphorus fractions at different sites; similarity percentage (SIMPER) analysis on the physicochemical data set showing the contribution of each variable to the differences between barren and developing, barren and mature, and developing and mature soil developmental stages in the Hailuogou chronosequence. Contribution (%) refers to the contribution of the single variables, and Cumulative (%) refers to the cumulative difference of all the variables considered. Download Table S1, XLSX file, 0.02 MB. Copyright © 2020 Bai et al. 2020 Bai et al. This content is distributed under the terms of the Creative Commons Attribution 4.0 International license.

### Changes in the *phoD* community composition along the chronosequence.

The rarefaction curves indicated that the *phoD* operational taxonomic units (OTUs) represented well the sampled populations (Fig. S3). At the barren and developing stages, the richness and diversity indices were higher in the rhizosphere than in the bulk soil; at the mature stage, the indices were higher in the bulk soil (Fig. S4).

10.1128/mSystems.00475-20.3 FIG S3 Rarefaction curves of the *phoD* sequences in samples from Hailuogou glacier chronosequence. S, rhizosphere; S1, <4 years old; S2, 22 years old; S3, 40 years old; S4, 54 years old; S5, 62 years old; S6, 89 years old; S7, 129 years old; T, bulk soil; T1, <4 years old; T2, 22 years old; T3, 40 years old; T4, 54 years old; T5, 62 years old;T6, 89 years old; T7, 129 years old. Download FIG S3, PDF file, 0.1 MB. Copyright © 2020 Bai et al. 2020 Bai et al. This content is distributed under the terms of the Creative Commons Attribution 4.0 International license.

10.1128/mSystems.00475-20.4 FIG S4 The diversity and richness of the *phoD* communities associated with the rhizosphere of *P. purdomii* and bulk soils along the Hailuogou glacier chronosequence. Asterisks indicate statistically significant differences (Kruskal-Wallis test, *P* < 0.05). Download FIG S4, PDF file, 0.2 MB. Copyright © 2020 Bai et al. 2020 Bai et al. This content is distributed under the terms of the Creative Commons Attribution 4.0 International license.

**FIG 1:**
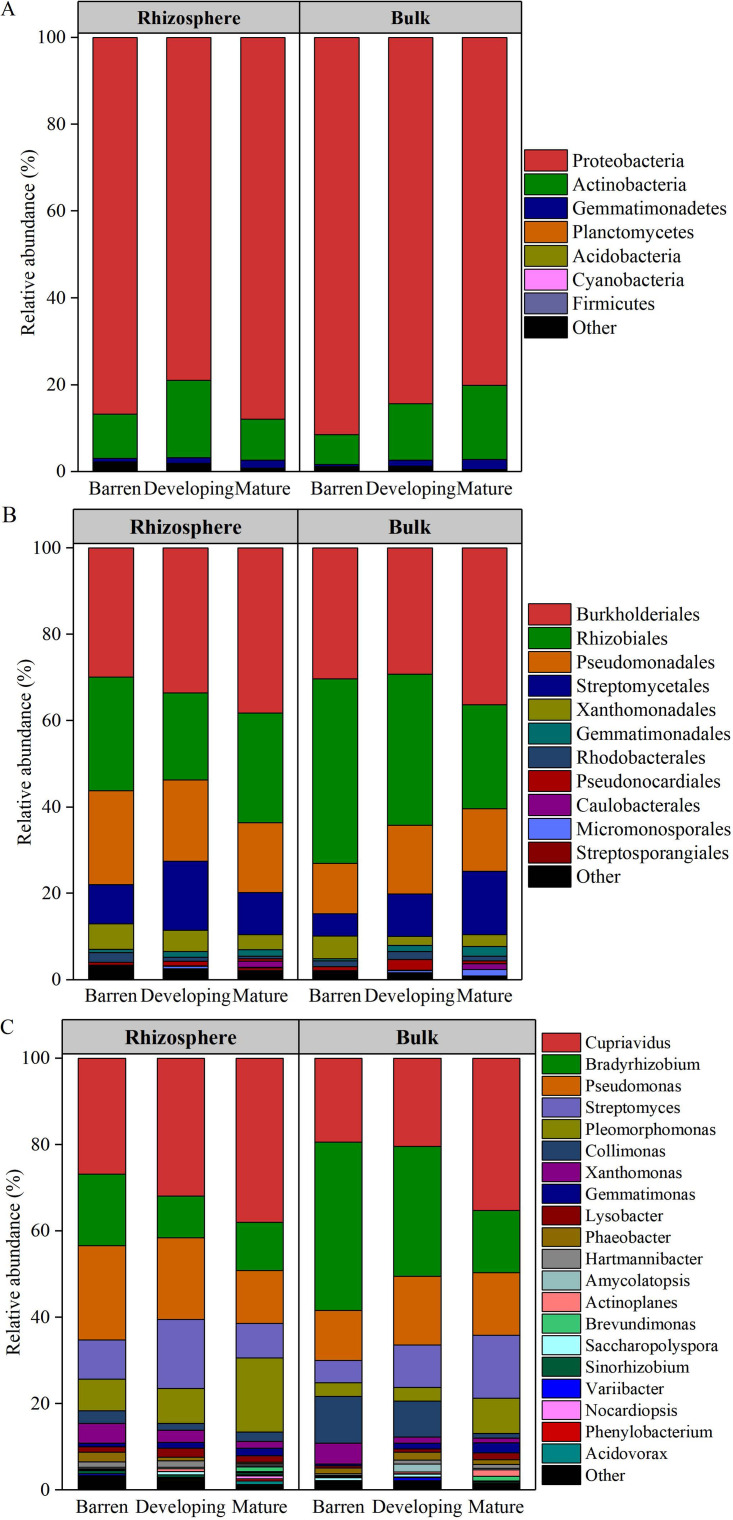
The *phoD* community composition in the rhizosphere of *P. purdomii* and bulk soils along the Hailuogou glacier chronosequence. (A) Phylum level; (B) order level; (C) genus level. Barren, 4- and 22-year-old soils; developing, 40- and 54-year-old soils; mature, 62-, 89-, and 129-year-old soils.

The *phoD* community composition changed along the soil developmental stages (Fig. 1). At the phylum level, the relative abundances of *Proteobacteria* (79 to 92%) and *Actinobacteria* (7 to 18%) were high both in the rhizosphere of *P. purdomii* and in the bulk soil at every stage (Table S2). In the rhizosphere, the relative abundance of *Proteobacteria* was lowest and that of the *Actinobacteria* was highest at the developing stage, whereas in the bulk soil, the relative abundance of *Proteobacteria* was highest and that of the *Actinobacteria* was lowest at the barren stage (*P* < 0.05) (Fig. 1A; Table S2).

10.1128/mSystems.00475-20.7 TABLE S2 The relative abundances of *phoD*-carrying taxa at the phylum, order, and genus levels along the Hailuogou glacier chronosequence. Download Table S2, XLSX file, 0.01 MB. Copyright © 2020 Bai et al. 2020 Bai et al. This content is distributed under the terms of the Creative Commons Attribution 4.0 International license.

At the order level, the relative abundance of *Burkholderiales* was highest (29 to 41%), followed by *Rhizobiales* (20 to 43%), *Pseudomonadales* (12 to 22%), and *Streptomycetales* (5 to 16%) (Fig. 1B). The relative abundance of *Burkholderiales* was highest at the mature stage in the rhizosphere (*P* < 0.05), that of *Rhizobiales* was higher at the barren stage than at the mature stage in the bulk soil, and that of *Pseudomonadales* was lowest at the mature stage in the rhizosphere (*P* < 0.05) (Fig. 1B; Table S2).

At the genus level, over 75% of the sequences were assigned into five genera: *Cupriavidus*, *Bradyrhizobium*, *Pseudomonas*, *Streptomyces*, and *Pleomorphomonas* (Fig. 1C). The relative abundances of *Cupriavidus* and *Pleomorphomonas* were higher and that of *Bradyrhizobium* was lower at the mature stage than at the barren stage (*P* < 0.05). In the rhizosphere, the relative abundance of *Pseudomonas* was lowest at the mature stage and that of *Streptomyces* was lowest at the developing stage (*P* < 0.05). In the bulk soil, the relative abundances of *Streptomyces* became higher along the soil development (*P* < 0.05) (Fig. 1C; Table S2).

In the bulk soil, the β-nearest taxon index (βNTI) distribution at the barren stage was consistent with variable selection (βNTI > +2) and with stochastic community assembly (−2 < βNTI < +2) at the later stages (Fig. S5). In the rhizosphere, the βNTI distribution was consistent with stochastic community assembly at all stages.

10.1128/mSystems.00475-20.5 FIG S5 The β-nearest taxon index (βNTI) distributions associated with the rhizosphere and bulk soils along the Hailuogou glacier chronosequence. Box plots of βNTI distributions across successional stages showing the median (thick black line), the first quartile (lower box bound), the third quartile (upper box bound), the range of data values that deviate from the box no more than 1.5 times the height of the box (vertical dashed lines), and outliers (open circles). Horizontal dashed lines indicate upper and lower significance thresholds at βNTI = +2 and −2, respectively. Download FIG S5, PDF file, 0.1 MB. Copyright © 2020 Bai et al. 2020 Bai et al. This content is distributed under the terms of the Creative Commons Attribution 4.0 International license.

### Characteristic taxa and distance decay in the *phoD* communities.

**FIG 2:**
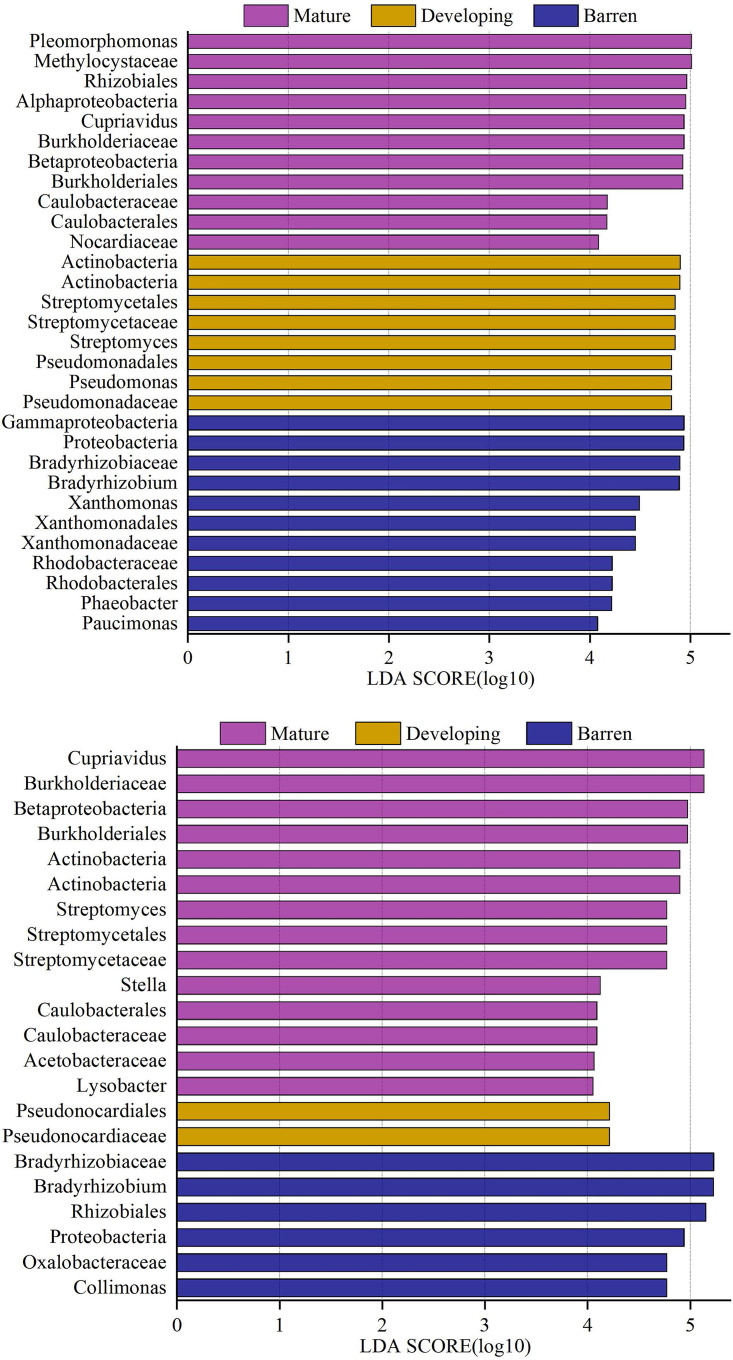
Taxa characterizing the differences between developmental stages in the rhizosphere of *P. purdomii* (top) and bulk soils (bottom) along the Hailuogou glacier chronosequence, identified using the linear discriminant analysis (LDA) effect size (LEfSe). Barren, 4- and 22-year-old soils; developing, 40- and 54-year-old soils; mature, 62-, 89-, and 129-year-old soils.

The linear discriminant analysis (LDA) effect size (LEfSe) analysis revealed that the taxa that characterized the differences between developmental stages belonged to *Proteobacteria*, *Actinobacteria*, and *Gemmatimonadetes* in the rhizosphere and to *Proteobacteria*, *Actinobacteria*, *Acidobacteria*, and *Gemmatimonadetes* in the bulk soil (Table S3). In the bulk soil and rhizosphere, 29 and 30 taxa, respectively, had large effect sizes with LDA scores of >4.0 (Fig. 2). OTUs assigned to *Pleomorphomonas* and *Methylocystaceae* in the mature-stage rhizosphere, *Bradyrhizobiaceae*, *Bradyrhizobium*, and *Rhizobiales* in the barren-stage bulk soil, and *Cupriavidus* and *Burkholderiaceae* in the mature-stage bulk soil had LDA scores of >5.0 (Fig. 2; Table S3).

10.1128/mSystems.00475-20.8 TABLE S3 Linear discriminant effect size (LEfSe) analysis of bacterial *phoD* OTUs in the rhizosphere and bulk soil in barren, developing, and mature soils along the Hailuogou chronosequence. Download Table S3, XLSX file, 0.02 MB. Copyright © 2020 Bai et al. 2020 Bai et al. This content is distributed under the terms of the Creative Commons Attribution 4.0 International license.

**FIG 3:**
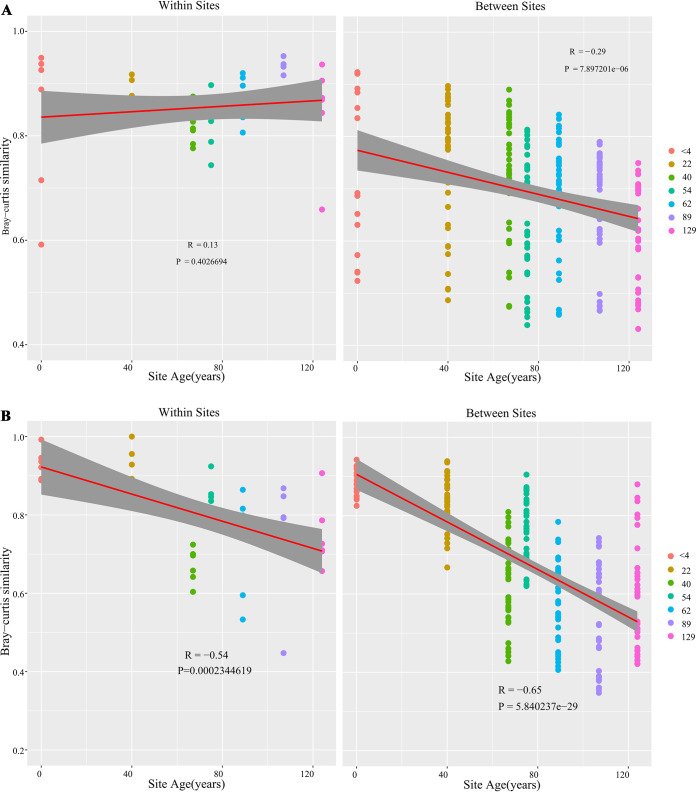
Bray-Curtis distance-based distance decay analysis of the *phoD* communities in the rhizosphere of *P. purdomii* (A) and bulk soils along the Hailuogou glacier chronosequence (B). Colored circles identify samples by age in years.

The within-plot Bray-Curtis similarity decreased in the bulk soil along the chronosequence, while it increased with soil developmental age in the rhizosphere (Fig. 3). When the similarity between the samples of different plots was plotted against the soil developmental stages, community similarity between distant samples decreased in both the bulk soil and rhizosphere (*P* < 0.0001). The rate of distance decay was higher in the bulk soils than in the rhizosphere (Fig. 3).

### Environmental parameters related to pedogenesis shape the *phoD* communities in rhizosphere and bulk soils.

**FIG 4:**
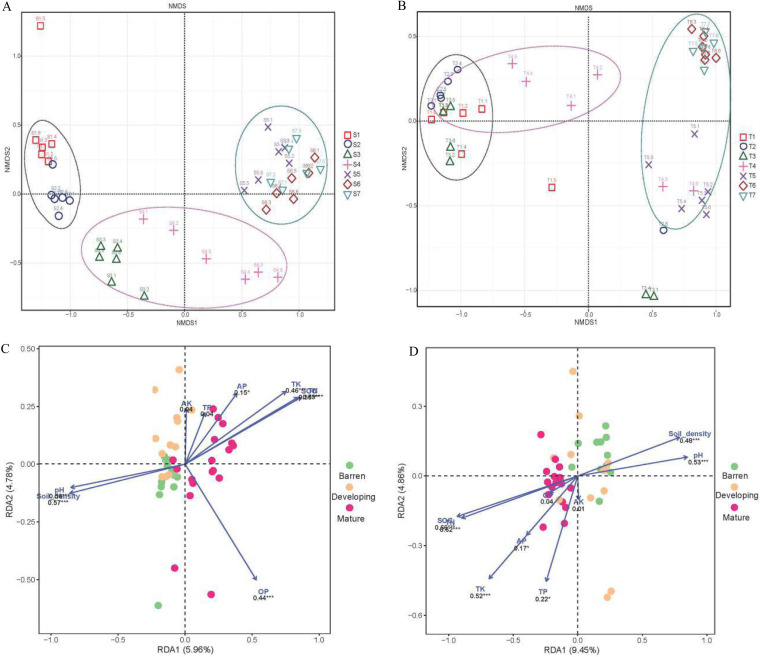
The clustering of *phoD* communities and the correlating soil physicochemical properties along the Hailuogou glacier chronosequence. (A and B) Nonmetric multidimensional scaling (NMDS) of the communities in the rhizosphere of *P. purdomii* (A) and bulk soils (B), based on unweighted UniFrac distances. Letter S indicates rhizosphere samples; S1, less than 4 years old; S2, 22 years old; S3, 40 years old; S4, 54 years old; S5, 62 years old; S6, 89 years old; S7, 129 years old. Letter T indicates bulk soil samples; T1, less than 5 years old; T2, 22 years old; T3, 40 years old; T4, 54 years old; T5, 62 years old; T6, 89 years old; T7, 129 years old. (C and D) Distance-based redundancy analysis (dbRDA) of the *phoD* communities (the percentages of 20 most abundant genera and summed percentage of other genera) and environmental factors in the rhizosphere (C) and bulk soil (D). SOC, soil organic carbon; TN, total nitrogen; TP, total phosphorus; AP, available phosphorus; TK, total potassium; AK, available potassium; CEC, cation exchange capacity; OP, organic phosphorus. * means significantly different at 0.05 level, ** is 0.01 level, and *** is 0.001 level.

In the nonmetric multidimensional scaling (NMDS) based on unweighted UniFrac distances, the rhizosphere soils clustered into three groups (Fig. 4A), corresponding to the barren, developing, and mature stages defined based on the soil physicochemical properties (Fig. S2). The bulk soil barren-stage and mature-stage samples were separated, but the developing-stage samples were scattered (analysis of similarities [ANOSIM], rhizosphere: *R* = 0.92, *P* < 0.001; bulk: *R* = 0.62, *P* < 0.001) (Fig. 4B).

Based on the distance-based redundancy analysis (dbRDA) data, in both the rhizosphere and bulk soil the differences in *phoD* community composition were related to differences in the bulk soil pH, density, and soil organic carbon (SOC), total nitrogen (TN), and total potassium (TK) contents (*P* < 0.001) (Fig. 4C and D; Table S4). In addition, the organic phosphorus (OP) content correlated with *phoD* community composition in the rhizosphere (*P* < 0.001) (Table S4), and total phosphorus (TP) content correlated with the *phoD* community composition in the bulk soil (*P* < 0.01) (Table S4).

10.1128/mSystems.00475-20.9 TABLE S4 The statistical significances of soil physicochemical properties in explaining the variation in *phoD* communities at genus level in the Hailuogou glacier chronosequence in the distance-based redundancy analysis (dbRDA). Download Table S4, PDF file, 0.05 MB. Copyright © 2020 Bai et al. 2020 Bai et al. This content is distributed under the terms of the Creative Commons Attribution 4.0 International license.

## DISCUSSION

Microorganisms that produce phosphatases play an important role in the cycling of phosphorus (P), a key nutrient in soil development. We studied the development, compositional turnover, and environmental drivers of microbial communities carrying phosphatase-encoding *phoD* in the course of primary succession in a glacier chronosequence. Mapelli et al. (18) analyzed microbial communities along the Midtre Lovénbreen glacier chronosequence and divided soil development into three stages including barren, developing, and mature soils. Based on soil properties, similar developmental stages were evident also along the Hailuogou glacier chronosequence. The soil pH values declined and the soil organic C (SOC) and total N (TN) contents increased along the chronosequence, plausibly due to increased litter production and increased aboveground biomass (5), which are known to result in root excretion of organic acids, accumulation of humic acids, and larger soil C and N pools (5, 19).

Compared to other major nutrients such as nitrogen or potassium, soil P has restricted mobility, which may make P a limiting nutrient in old soils (20, 21). However, in our study the total P content and the concentrations of all the P forms were lower in the barren soil than in the developing and mature soil. Furthermore, the available P (AP)-to-total P (TP) ratios were two to almost five times higher in the developing and mature soils than in the barren soil. The availability of P is generally governed by pH, with maximum availabilities at approximately pH 4.5 and 6.5 (22). However, in our study the AP-to-TP ratio was higher at the 89-year-old site where pH was 5.4 than at the 54-, 62-, and 129-year-old sites where pH values were closer to the optimum for P availability. P availability is also related to phosphatase biosynthesis by both plant roots and microorganisms and phosphatase activity (23, 24). Possibly, the high soil C content that is known to affect microbial growth (25) enhanced phosphatase biosynthesis and activity, resulting in a high AP-to-TP ratio at the 89-year-old site. In the barren soil, both pH and low levels of phosphatase biosynthesis by plants and microorganisms due to the smaller plant cover (5) and lower soil C contents may have affected the availability of P.

Variation of the microbial community during primary succession is a dynamic and complex process, including increases in microbial abundance and diversity and changes in the community composition (4). In a High Arctic glacier chronosequence, the bacterial community composition changed along the soil development gradient, and dominant taxa such as *Acidobacteria* and *Chloroflexi* were enriched and proposed to play relevant functional roles across the gradient (18). Our results revealed that the *phoD* community composition and diversity changed along the developmental degree, and the taxon distribution patterns were different in rhizosphere and bulk soils. Possibly, the changes in the *phoD* community composition in both rhizosphere and bulk soil reflected the increased aboveground vegetation biomass along the chronosequence (5). Except at the barren stage in bulk soil, the βNTI distribution was consistent with stochastic community assembly, expected of resource-rich environments due to low competitive pressure (26, 27). In the bulk soil, the resources were evidenced as the higher SOC and TN contents at developing and mature stages. In the rhizosphere, root exudates may have provided the resources (28, 29). At the barren stage in bulk soil, the βNTI distribution was consistent with variable selection, possibly due to spatial variation in the selective environment, which may result in more than expected variation in the community composition (27).

Across diverse soil and climatic conditions, *Actinobacteria*, *Cyanobacteria*, *Firmicutes*, *Planctomycetes*, and *Proteobacteria* have been identified as the key *phoD*-harboring phyla (11, 30). In our study, the taxa characterizing the differences between developmental stages were assigned to *Proteobacteria* and *Actinobacteria*; more specifically, to *Pseudomonadales* in the rhizosphere and *Bradyrhizobium* and *Cupriavidus* in the bulk soil. Nitrogen-fixing organisms colonized SOC- and N-poor soils that typically characterize early stages of succession, promoted the accumulation of soil N, and facilitated colonization by later successional species and plant establishment and growth (31–33). In our study, the relative abundances of the N_2_-fixing *Bradyrhizobium* and *Cupriavidus* (34, 35) were higher in the early stage and in the later stage, respectively. *Pseudomonas* bacteria that have plant growth promotion abilities (36, 37) were of high relative abundance in the rhizosphere at the early stages. The relative abundance of *Streptomyces* bacteria that are capable of degrading organic C sources (38) became higher along the succession, possibly due to the higher SOC content. Thus, the increasing nutrient and plant development resulted in different key taxonomic groups at the three stages along the chronosequence.

Community similarity decline with increasing geographic distance, i.e., distance decay, has been proposed to indicate locally adapted species or dispersal limitation (39). In this study, the within-site similarity in *phoD* community composition decreased in the bulk soils along the chronosequence, and both the rhizosphere and bulk soil community similarities decreased with increasing distance. Similar to the work of Mapelli et al. (18), the distance decay rate was higher in the bulk soils than in the rhizosphere, showing that plants affected the selection of rhizosphere microbiota and shaped the rhizosphere community assembly. Plausibly, the rhizospheres along the chronosequence were more similar to each other than they were to the bulk soils. Overall, the distance decay implied dispersal limitation.

Soil pH and SOC and TN contents are considered the most prevailing ecological drivers for bacterial communities (5, 11). Studies across other deglaciated chronosequences have shown that changes in soil nutrient and C contents affect bacterial community composition and function (40, 41). For rhizosphere bacterial communities in the Damma Glacier forefield, Edwards et al. (41) found that SOC and mineral N were dominant factors affecting bacterial communities across the chronosequence. In Peru, during the early-stage primary succession after glacial retreat, microbial primary producers were limited by P (9). According to a previous survey, the P availability may become a limited resource for microbial activities in the Hailuogou glacier chronosequence (42). In our study, the soil properties were measured from the bulk soil and may differ from the rhizosphere soil, e.g., C content may be higher and P content lower (43). However, our results indicated that bulk soil density, SOC, TN, pH, and TK were the most important factors among soil properties for both the bulk soil and rhizosphere *phoD* communities. The increasing aboveground biomass along the chronosequence (5) may have directly contributed to the differences in SOC, TN, and pH.

In summary, we selected the pioneer species *Populus purdomii* Rehder as a model plant to study the *phoD* communities in rhizosphere and bulk soils along the Hailuogou glacier forefield chronosequence. Changes in the soil properties were accompanied with changes in the *phoD* community along the chronosequence. The differences in the *phoD* community composition were mostly related to differences in soil pH and SOC and TN contents.

## MATERIALS AND METHODS

### Study sites.

The Hailuogou glacier is located at the Gongga Mountain (29°34′07.83″N, 101°59′40.74″E, 7,556 m above sea level [MASL]) on the southeastern edge of the Tibetan Plateau. The recession of the Hailuogou glacier has been occurring since the Little Ice Age, and the glacier melting has accelerated continuously. The 2-km-long glacier forefield extends to the northeast and has an elevation difference of 100 m. In Hailuogou the mean annual precipitation is 2,000 mm, most rainfall occurs between June and October (44), and the annual mean temperature is 3.8°C, ranging from −4.3°C in January to 11.9°C in July. The sampling sites were on a seven-scale chronosequence with approximate ages, i.e., numbers of successional years after glacier retreat, calibrated as described earlier (see Fig. S1 in the supplemental material) (5).

10.1128/mSystems.00475-20.1 FIG S1 Location of the Hailuogou chronosequence and sampling sites. 1, <4 years since glacier retreat (T1); 2, 22 years since glacier retreat (T2); 3, 40 years since glacier retreat (T3); 4, 54 years since glacier retreat (T4); 5, 62 years since glacier retreat (T5); 6, 89 years since glacier retreat (T6); 7, 129 years since glacier retreat (T7). Download FIG S1, PDF file, 0.1 MB. Copyright © 2020 Bai et al. 2020 Bai et al. This content is distributed under the terms of the Creative Commons Attribution 4.0 International license.

*P. purdomii* trees were present at every primary successional stage (Table S1) and therefore selected as the model plant in this study. Approximately 5 years after glacier retreat, the patches of bare land composed of the heaps of weathering rocks were colonized by herbs, mainly Astragalus adsurgens, and seedlings of Hippophae rhamnoides and *P. purdomii*. The first trees, mainly *H. rhamnoides*, *Salix* spp., and *P. purdomii*, were present at the youngest site and visually dominated the 22-year-old site. *H. rhamnoides* and *P. purdomii* dominated the 40-year-old site, creating a closed canopy that shades nearly the entire soil surface and a thick layer of litter. The forest at the 54-year-old site was mainly composed of *P. purdomii*, and that at the 62-year-old site was composed of *P. purdomii*, Betula utilis, and Abies fabri. At the 89-year-old site *P. purdomii* was gradually replaced by *A. fabri* and Picea brachytyla. The last stage spans from 89-year-old (not included) to 129-year-old climax vegetation communities, with *P. brachytyla* and *A. fabri* as the dominant species, and sporadically distributed *P. purdomii* (42).

### Sampling.

Along the seven-scale chronosequence, six 20- by 20-m experimental plots with at least 20 m of distance between the plots were established on 10 August 2018, except in stages 1 and 2 where 10- by 10-m plots with approximately 10 m of distance between the plots were used due to the smaller area. Five soil cores were collected from the center and each corner of the quadrat using a 5-cm-diameter soil corer after removal of litter from soil surface by hand. The five soil cores were combined as one composite soil sample. In total, we analyzed 42 samples (6 replicate samples × 7 sites) from the rhizosphere and 42 samples from the bulk soil. The lateral roots were removed from the soil by using a clean spade and gently shaken to remove the soil not tightly attached to the roots; the soil still adhering to the roots was defined as the rhizosphere soil. Roots were put into sterile plastic bags and transported to the lab. The rhizosphere soil was separated from the roots by moderate agitation in 50 ml of sterile 0.9% NaCl solution for 5 min, followed by centrifugation at 8,000 × *g* for 10 min (45). Then, the three repetitions of rhizosphere soil in each plot were mixed thoroughly as one composite sample. Bulk soil with no contact with the root system of *P. purdomii* or other visible plants at 50 to 100 cm of distance from the sampled plants was aseptically sampled from 5- to 20-cm depth after removing the 1- to 5-cm-thick plant litter layers and put on ice for transporting. After the soil samples were transported to the laboratory, the bulk soils were divided into two subparts, and one was used for soil physicochemical analyses, while the other part, together with rhizosphere soils, was stored at −20°C for molecular analysis.

### Soil physicochemical analyses.

Soil pH and soil organic carbon (SOC), total nitrogen (TN), total phosphorus (TP), available P (AP), and available potassium (AK) contents were analyzed as described previously (46). The soil density, dissolved organic carbon (DOC), cation exchange capacity (CEC), exchangeable cations (Ca_exc_, Mg_exc_, K_exc_, Na_exc_), total Ca, Mg, K, and Na contents, and soil respiration were determined as described previously (46, 47). Soil P fractions were analyzed using a modified sequential extraction technique (48, 49).

### DNA extraction, *phoD* gene amplification, and sequencing.

Total DNA was extracted from 0.5 g of soil using the Fast DNA Spin kit for Soil (MP Inc., CA, USA) following the manufacturer’s instructions and stored at −20°C. Primer pair ALPS-F730 (CAGTGGGACGACCACGAGGT) and ALPS-R1101 (GAGGCCGATCGGCATGTCG) with adapter and barcode sequences was applied to amplify the *phoD* gene (50). Amplification was done in a 25-μl volume containing 1× MyTaq reaction buffer (including MgCl_2_ and deoxynucleoside triphosphates [dNTPs]), 0.5 μM (each) primer, and 0.6 U of MyTaq polymerase (Bioline, NSW, Australia) with 10 ng DNA as the template. The amplification program in an S1000 thermocycler (Bio-Rad Laboratories, CA, USA) included an initial denaturation for 5 min at 95°C, followed by 35 cycles of denaturation for 30 s at 95°C, annealing for 30 s at 57°C, and extension for 30 s at 72°C, and final extension for 5 min at 72°C. The PCR products from 3 replicate amplifications per sample were pooled and purified with the AxyPrep DNA purification kit (Axygen Biotech, Hangzhou, China) and quantified using Promega QuantiFluor (Invitrogen, Carlsbad, CA, USA). Purified amplicons were pooled in equimolar concentrations and sequenced using MiSeq reagent kit V2 on an Illumina MiSeq platform (Illumina Biotech, CA, USA).

### Bioinformatics analysis.

The sequence data were processed using the QIIME pipeline (51). Reads with an average quality score below 20 were filtered out, and the remaining reads were trimmed at 200 bp and 450 bp as minimum and maximum length, respectively. Sequences were clustered into operational taxonomic units (OTUs) using the UCLUST sequence alignment tool at 97% sequence similarity (52), and the representative sequences of OTUs were assigned to taxa using the Ribosomal Database Project classifier (53). Alpha diversity indices, i.e., Shannon index for diversity and Chao1 for richness, were calculated based on the number of OTUs and their relative abundances using QIIME2 (51). Rarefaction curves were generated using QIIME2 (51). The sequences were rarefied to depths of 15,018 and 15,040 sequences per sample for bulk soil and rhizosphere, respectively.

### Statistical analysis.

The sites were grouped into developmental stages based on soil physicochemical properties using the Canonical Analysis of Principal coordinates (CAP) (Fig. S2) in R package BiodiversityR as described earlier (18). Most important chemical variables defining the developmental stages were retrieved using similarity percentage analysis (SIMPER). Differences in the alpha diversity indices were tested using two-way ANOVA. Differences were considered statistically significant at *P* < 0.05. Differences in the relative abundances of *phoD*-carrying taxa at phylum, order, and genus levels were tested using ANOVA.

Beta diversity of the *phoD* communities, based on unweighted UniFrac distances, was visualized using nonmetric multidimensional scaling (NMDS) in the R package vegan (54, 55). The variation in *phoD* communities at genus level explained by the soil properties was analyzed using distance-based redundancy analysis (dbRDA) in the R package vegan in R (54, 55). For the dbRDA, the *phoD* community matrix included the percentages of the 20 most abundant genera and summed percentage of other genera. The dbRDA model was statistically tested using the permutest function in the vegan package with 999 permutations. The goodness of fit for each operational parameter was estimated by applying the envfit function (999 permutations). Differences in the rate of distance decay of community similarity (Bray-Curtis) between the bulk and rhizosphere soil along the soil developmental stages were tested with an analysis of covariance. The statistical significance of compositional differences of *phoD* communities along the soil developmental stages was tested with analysis of similarities (ANOSIM). The β-nearest taxon index (βNTI) was calculated for the soil fractions and developmental stages. βNTIs smaller than −2 and greater than 2 were interpreted as indicating the dominance of homogeneous and variable selection, respectively (27). Taxa that characterized the differences between developmental stages were identified using the linear discriminant analysis (LDA) effect size (LEfSe) method on the Galaxy online platform (https://huttenhower.sph.harvard.edu/galaxy/) (56).

### Data availability.

The DNA-Seq data (fastq files) are publicly available in National Center for Biotechnology Information (NCBI) under accession no. PRJNA597270.

## References

[1] Odum EP 1969 The strategy of ecosystem development. Science 164:262–270. doi:10.1126/science.164.3877.262. 5776636

[2] Ortiz-Álvarez R, Fierer N, Ríos ADL, Casamayor EO, Barberán A 2018 Consistent changes in the taxonomic structure and functional attributes of bacterial communities during primary succession. ISME J 12:1658–1667. doi:10.1038/s41396-018-0076-2. 29463893 PMC6018800

[3] Jumpponen A, Brown SP, Trappe JM, Cazares E, Strommer R 2012 Twenty years of research on fungal-plant interactions on Lyman Glacier forefront—lessons learned and questions yet unanswered. Fungal Ecol 5:430–442. doi:10.1016/j.funeco.2012.01.002.

[4] Brown SP, Jumpponen A 2014 Contrasting primary successional trajectories of fungi and bacteria in retreating glacier soils. Mol Ecol 23:481–497. doi:10.1111/mec.12487. 24112459

[5] Jiang YL, Lei YB, Yang Y, Korpelainen H, Niinemets Ü, Li CY 2018 Divergent assemblage patterns and driving forces for bacterial and fungal communities along a glacier forefield chronosequence. Soil Biol Biochem 118:207–216. doi:10.1016/j.soilbio.2017.12.019.

[6] Knelman JE, Legg TM, O’Neill SP, Washenberger CL, González A, Cleveland CC, Nemergut DR 2012 Bacterial community structure and function change in association with colonizer plants during early primary succession in a glacier forefield. Soil Biol Biochem 46:172–180. doi:10.1016/j.soilbio.2011.12.001.

[7] Zhou J, Wu Y, Prietzel J, Bing HJ, Yu D, Sun SQ, Luo J, Sun HY 2013 Changes of soil phosphorus speciation along a 120-year soil chronosequence in the Hailuogou Glacier retreat area (Gongga Mountain, SW China). Geoderma 195-196:251–259. doi:10.1016/j.geoderma.2012.12.010.

[8] Coomes DA, Bentley WA, Tanentzap AJ, Burrows LE 2013 Soil drainage and phosphorus depletion contribute to retrogressive succession along a New Zealand chronosequence. Plant Soil 367:77–91. doi:10.1007/s11104-013-1649-5.

[9] Darcy JL, Schmidt SK, Knelman JE, Cleveland CC, Castle SC, Nemergut DR 2018 Phosphorus, not nitrogen, limits plants and microbial primary producers following glacial retreat. Sci Adv 4:eaaq0942. doi:10.1126/sciadv.aaq0942. 29806022 PMC5966225

[10] Ragot SA, Kertesz MA, Bünemann EK 2015 *phoD* alkaline phosphatase gene diversity in soil. Appl Environ Microbiol 81:7281–7289. doi:10.1128/AEM.01823-15. 26253682 PMC4579420

[11] Ragot SA, Huguenin-Elie O, Kertesz MA, Frossard E, Bünemann EK 2016 Total and active microbial communities and *phoD* as affected by phosphate depletion and pH in soil. Plant Soil 408:15–30. doi:10.1007/s11104-016-2902-5.

[12] Luo H, Benner R, Long RA, Hu J 2009 Subcellular localization of marine bacterial alkaline phosphatases. Proc Natl Acad Sci U S A 106:21219–21223. doi:10.1073/pnas.0907586106. 19926862 PMC2795515

[13] Cui H, Zhou Y, Gu Z, Zhu H, Fu S, Yao Q 2015 The combined effects of cover crops and symbiotic microbes on phosphatase gene and organic phosphorus hydrolysis in subtropical orchard soils. Soil Biol Biochem 82:119–126. doi:10.1016/j.soilbio.2015.01.003.

[14] Zhong XH, Zhang WJ, Luo J 1999 The characteristics of the mountain ecosystem and environment in the Gongga Mountain region (in Chinese with English abstract). Ambio 28:648–654.

[15] He L, Tang Y 2008 Soil development along primary succession sequences on moraines of Hailuogou glacier, Gongga Mountain, Sichuan, China. Catena 72:259–269. doi:10.1016/j.catena.2007.05.010.

[16] Yang Y, Wang GX, Shen HH, Yang Y, Cui HJ, Liu Q 2014 Dynamics of carbon and nitrogen accumulation and C: N stoichiometry in a deciduous broadleaf forest of deglaciated terrain in the eastern Tibetan Plateau. Forest Ecol Manag 312:10–18. doi:10.1016/j.foreco.2013.10.028.

[17] Zhou J, Wu YH, Turner BL, Sun HY, Wang JP, Bing HJ, Luo J, He XL, Zhu H, He QQ 2019 Transformation of soil organic phosphorus along the Hailuogou post-glacial chronosequence, southeastern edge of the Tibetan Plateau. Geoderma 352:414–421. doi:10.1016/j.geoderma.2019.05.038.

[18] Mapelli F, Marasco R, Fusi M, Fusi M, Scaglia B, Tsiamis G, Rolli E, Fodelianakis S, Bourtzis K, Ventura S, Tambone F, Adani F, Borin S, Daffonchio D 2018 The stage of soil development modulates rhizosphere effect along a High Arctic desert chronosequence. ISME J 12:1188–1198. doi:10.1038/s41396-017-0026-4. 29335640 PMC5931989

[19] Lauber CL, Hamady M, Knight R, Fierer N 2009 Pyrosequencing-based assessment of soil pH as a predictor of soil bacterial community structure at the continental scale. Appl Environ Microbiol 75:5111–5120. doi:10.1128/AEM.00335-09. 19502440 PMC2725504

[20] Leonardo D, Anibal DM, Vincent M 2018 Environmental drivers of soil phosphorus composition in natural ecosystems. Biogeosciences 15:4575–4595. doi:10.5194/bg-15-4575-2018.

[21] Hinsinger P, Herrmann L, Lesueur D, Robin A, Trap J, Waithaisong K, Plassard C 2015 Impact of roots, microorganisms and microfauna on the fate of soil phosphorus in the rhizosphere. Annu Plant Rev 48:377–407. doi:10.1002/9781119312994.apr0528.

[22] Penn CJ, Camberato JJ 2019 A critical review on soil chemical processes that control how soil pH affects phosphorus availability to plants. Agriculture 9:120–118. doi:10.3390/agriculture9060120.

[23] Spohn M, Treichel NS, Cormann M, Schloter M, Fischer D 2015 Distribution of phosphatase activity and various bacterial phyla in the rhizosphere of Hordeum vulgare L. depending on P availability. Soil Biol Biochem 89:44–51. doi:10.1016/j.soilbio.2015.06.018.

[24] Hofmann K, Heuck C, Spohn M 2016 Phosphorus resorption by young beech trees and soil phosphatase activity as dependent on phosphorus availability. Oecologia 181:369–379. doi:10.1007/s00442-016-3581-x. 26875186

[25] Göransson H, Venterink HO, Bååth E 2011 Soil bacterial growth and nutrient limitation along a chronosequence from a glacier forefield. Soil Biol Biochem 43:1333–1340. doi:10.1016/j.soilbio.2011.03.006.

[26] Chase JM 2010 Stochastic community assembly causes higher biodiversity in more productive environments. Science 328:1388–1391. doi:10.1126/science.1187820. 20508088

[27] Dini-Andreote F, Stegen JC, Dirk van Elsas J, Salles JF 2015 Disentangling mechanisms that mediate the balance between stochastic and deterministic processes in microbial succession. Proc Natl Acad Sci U S A 112:1326–1332. doi:10.1073/pnas.1414261112. PMC4371938 25733885

[28] Badri DV, Chaparro JM, Zhang R, Shen Q, Vivanco JM 2013 Application of natural blends of phytochemicals derived from the root exudates of Arabidopsis to the soil reveal that phenolic-related compounds predominantly modulate the soil microbiome. J Biol Chem 288:4502–4512. doi:10.1074/jbc.M112.433300. 23293028 PMC3576057

[29] Chaparro JM, Badri DV, Bakker MG, Sugiyama A, Manter DK, Vivanco JM 2013 Root exudation of phytochemicals in Arabidopsis follows specific patterns that are developmentally programmed and correlate with soil microbial functions. PLoS One 8:e55731. doi:10.1371/journal.pone.0055731. 23383346 PMC3562227

[30] Ragot SA, Kertesz MA, Mészáros E, Frossard E, Bünemann EK 2017 Soil *phoD* and *phoX* alkaline phosphatase gene diversity responds to multiple environmental factors. FEMS Microbiol Ecol 93:fiw212. doi:10.1093/femsec/fiw212. 27737901

[31] Nemergut DR, Anderson SP, Cleveland CC, Martin AP, Miller AE, Seimon A, Schmidt SK 2007 Microbial community succession in an unvegetated, recently deglaciated soil. Microb Ecol 53:110–122. doi:10.1007/s00248-006-9144-7. 17186150

[32] Walker LR, Clarkson BD, Silvester WB, Clarkson BR 2003 Colonization dynamics and facilitative impacts of a nitrogen-fixing shrub in primary succession. J Veg Sci 14:277–290. doi:10.1111/j.1654-1103.2003.tb02153.x.

[33] Carrillo-Castañeda G, Juárez Muños J, Peralta-Videa JR, Gomez E, Tiemannb KJ, Duarte-Gardea M, Gardea-Torresdey JL 2002 Alfalfa growth promotion by bacteria grown under iron limiting conditions. Adv Environ Res 6:391–399. doi:10.1016/S1093-0191(02)00054-0.

[34] Agarwa L, Prakash A, Purohit HJ 2019 Expression of autotrophic genes under CO_2_ environment and genome mining of desert bacterium *Cupriavidus* sp. HPC (L). Bioresour Technol Rep 7:100258–100267. doi:10.1016/j.biteb.2019.100258.

[35] Wang Q, Quensen JF III, Fish JA, Lee TK, Sun YN, Tiedje JM, Cole JR 2013 Ecological patterns of *nifH* genes in four terrestrial climatic zones explored with targeted metagenomics using frameBot, a new informatics tool. mBio 4:e00592-13. doi:10.1128/mBio.00592-13. 24045641 PMC3781835

[36] Zamioudis C, Pieterse C 2012 Modulation of host immunity by beneficial microbes. Mol Plant Microbe Interact 25:139–150. doi:10.1094/MPMI-06-11-0179. 21995763

[37] Philippot L, Raaijmakers JM, Lemanceau P, Putten W 2013 Going back to the roots: the microbial ecology of the rhizosphere. Nat Rev Microbiol 11:789–799. doi:10.1038/nrmicro3109. 24056930

[38] Sun Z, Lv Y, Liu Y, Ren R 2016 Removal of nitrogen by heterotrophic nitrification—aerobic denitrification of a novel metal resistant bacterium *Cupriavidus* sp. S1. Bioresour Technol 220:142–150. doi:10.1016/j.biortech.2016.07.110. 27566522

[39] Bryant JA, Lamanna C, Morlon H, Kerkhoff AJ, Enquist BJ, Green JL 2008 Microbes on mountainsides: contrasting elevational patterns of bacterial and plant diversity. Proc Natl Acad Sci U S A 105:11505–11511. doi:10.1073/pnas.0801920105. 18695215 PMC2556412

[40] Tscherko D, Hammesfahr U, Marx MC, Kandeler E 2004 Shifts in rhizosphere microbial communities and enzyme activity of Poa alpina across an alpine chronosequence. Soil Biol Biochem 36:1685–1698. doi:10.1016/j.soilbio.2004.07.004.

[41] Edwards IP, Bürgmann H, Miniaci C, Zeyer J 2006 Variation in microbial community composition and culturability in the rhizosphere of *Leucanthemopsis alpina* (L.) Heywood and adjacent bare soil along an alpine chronosequence. Microb Ecol 52:679–692. doi:10.1007/s00248-006-9097-x. 16909346

[42] Zhou J, Bing H, Wu Y, Sun H, Wang J 2018 Weathering of primary mineral phosphate in the early stages of ecosystem development in the Hailuogou Glacier foreland chronosequence. Eur J Soil Sci 69:450–461. doi:10.1111/ejss.12536.

[43] Hinsinger P, Gobran GR, Gregory PJ, Wenzel WW 2005 Rhizosphere geometry and heterogeneity arising from root-mediated physical and chemical processes. New Phytol 168:293–303. doi:10.1111/j.1469-8137.2005.01512.x. 16219069

[44] Wang J, Wu Y, Zhou J, Bing H, Sun H 2016 Carbon demand drives microbial mineralization of organic phosphorus during the early stage of soil development. Biol Fertil Soils 52:825–839. doi:10.1007/s00374-016-1123-7.

[45] Marilley L, Vogt G, Blanc M, Aragno M 1998 Bacterial diversity in the bulk soil and rhizosphere fractions of *Lolium perenne* and *Trifolium repens* as revealed by PCR restriction analysis of 16S rDNA. Plant Soil 198:219–224. doi:10.1023/A:1004309008799.

[46] Borin S, Ventura S, Tambone F, Mapelli F, Schubotz F, Brusetti L, Scaglia B, D’Acqui LP, Solheim B, Turicchia S, Marasco R, Hinrichs K-U, Baldi F, Adani F, Daffonchio D 2010 Rock weathering creates oases of life in a High Arctic desert. Environ Microbiol 12:293–303. doi:10.1111/j.1462-2920.2009.02059.x. 19840107

[47] Scaglia B, Pognani M, Adani F 2015 Evaluation of hormone-like activity of the dissolved organic matter fraction (DOM) of compost and digestate. Sci Total Environ 514:314–321. doi:10.1016/j.scitotenv.2015.02.009. 25668284

[48] Ruttenberg KC 1992 Development of a sequential extraction method for different forms of phosphorus in marine-sediments. Limnol Oceanogr 37:1460–1482. doi:10.4319/lo.1992.37.7.1460.

[49] Turner BL, Newman S 2005 Phosphorus cycling in wetland soils: the importance of phosphate diesters. J Environ Qual 34:1921–1929. doi:10.2134/jeq2005.0060. 16151243

[50] Sakurai M, Wasaki J, Tomizawa Y, Shinano T, Osaki M 2008 Analysis of bacterial communities on alkaline phosphatase genes in soil supplied with organic matter. Soil Sci Plant Nutr 54:62–71. doi:10.1111/j.1747-0765.2007.00210.x.

[51] Caporaso JG, Lauber CL, Walters WA, Berg-Lyons D, Huntley J, Fierer N, Owens SM, Betley J, Fraser L, Bauer M, Gormley N, Gilbert JA, Smith G, Knight B 2012 Ultra-high-throughput microbial community analysis on the Illumina HiSeq and MiSeq platforms. ISME J 6:1621–1624. doi:10.1038/ismej.2012.8. 22402401 PMC3400413

[52] Edgar RC 2010 Search and clustering orders of magnitude faster than BLAST. Bioinformatics 26:2460–2461. doi:10.1093/bioinformatics/btq461. 20709691

[53] Caporaso JG, Kuczynski J, Stombaugh J, Bittinger K, Bushman FD, Costello EK, Fierer N, Peña AG, Goodrich JK, Gordon JI, Huttley GA, Kelley ST, Knights D, Koenig E, Ley RE, Lozupone CA, McDonald D, Muegge BD, Pirrung M, Reeder J, Sevinsky JR, Turnbaugh PJ, Walters WA, Widmann J, Yatsunenko T, Zaneveld J, Knight R, 2010 QIIME allows analysis of high-throughput community sequencing data. Nat Methods 7:335–336. doi:10.1038/nmeth.f.303. 20383131 PMC3156573

[54] Pollard KS, Gilbert HN, Ge Y, Taylor S, Dudoit S 2010 Multtest: resampling-based multiple hypothesis testing. R package version 2.17.0. R Development Core Team. R: a language and environment for statistical computing. https://cran.r-project.org.

[55] Oksanen J, Blanchet FG, Kindt R, Pierre L, Minchin PR, O’Hara RB, Simpson GL, Solymos P, Stevens MHH, Wagner H 2016 Vegan: community ecology package. R package version 2.4-4. https://CRAN.R-project.org/package=vegan.

[56] Segata N, Huttenhower C, Waldron L, Garrett WS, Gevers D, Izard J, Miropolsky L 2011 Metagenomic biomarker discovery and explanation. Genome Biol 12:R60. doi:10.1186/gb-2011-12-6-r60. 21702898 PMC3218848

