# Supplementary figures and images for: Plant and Soil Development Cooperatively Shaped the Composition of the phoD-Harboring Bacterial Community along the Primary Succession in the Hailuogou Glacier Chronosequence
Source: mSystems. 2020 Jul 28;5(4):e00475-20. doi: 10.1128/mSystems.00475-20 (PMC7394357; doi:10.1128/mSystems.00475-20)

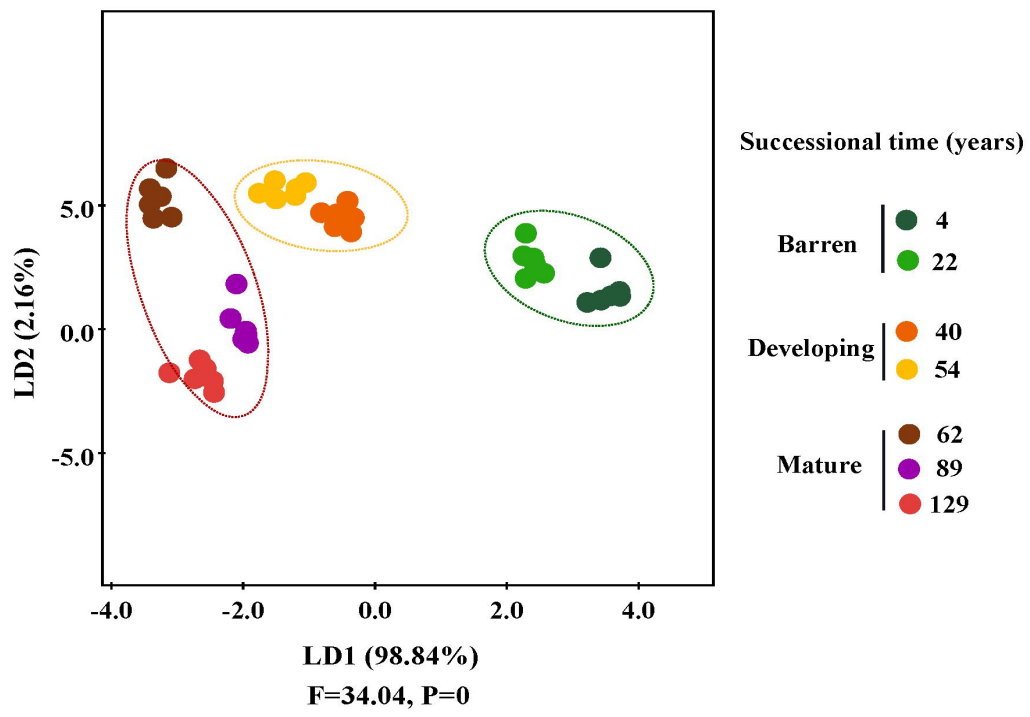

Supplement: FIG S2 [file mSystems.00475-20-sf002.pdf]

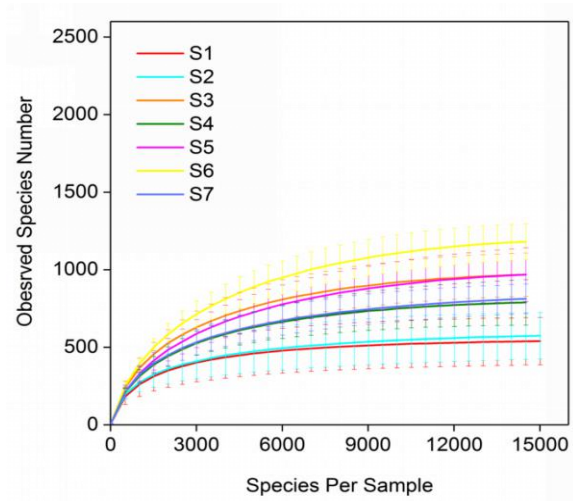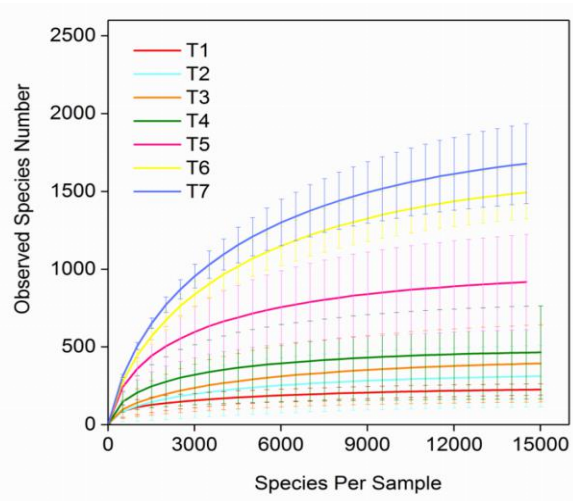

Supplement: FIG S3 [file mSystems.00475-20-sf003.pdf]

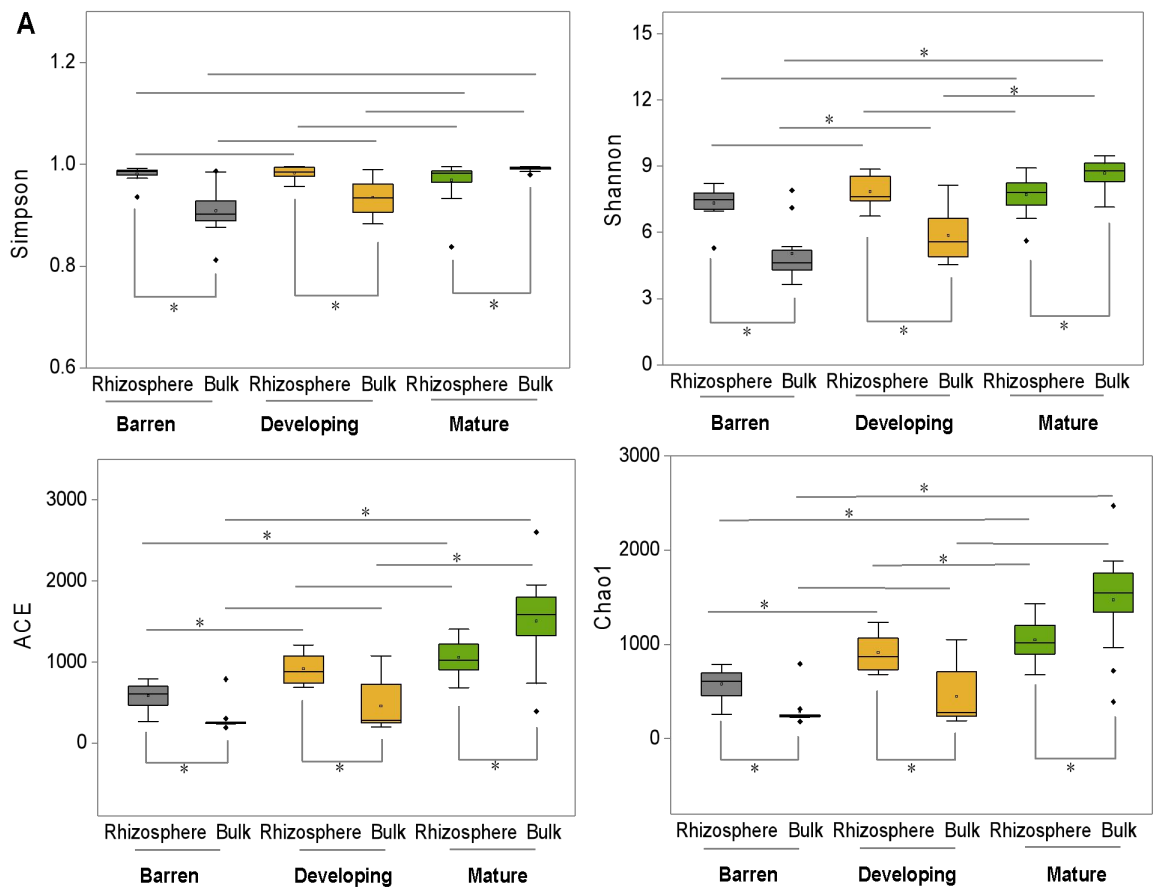

Supplement: FIG S4 [file mSystems.00475-20-sf004.pdf]

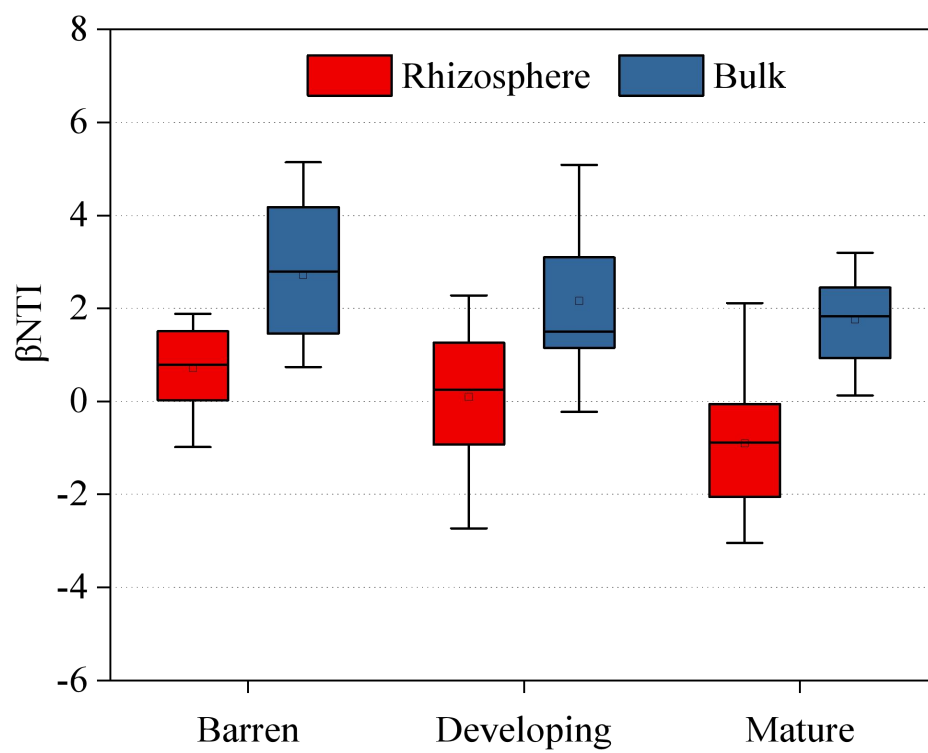

Supplement: FIG S5 [file mSystems.00475-20-sf005.pdf]

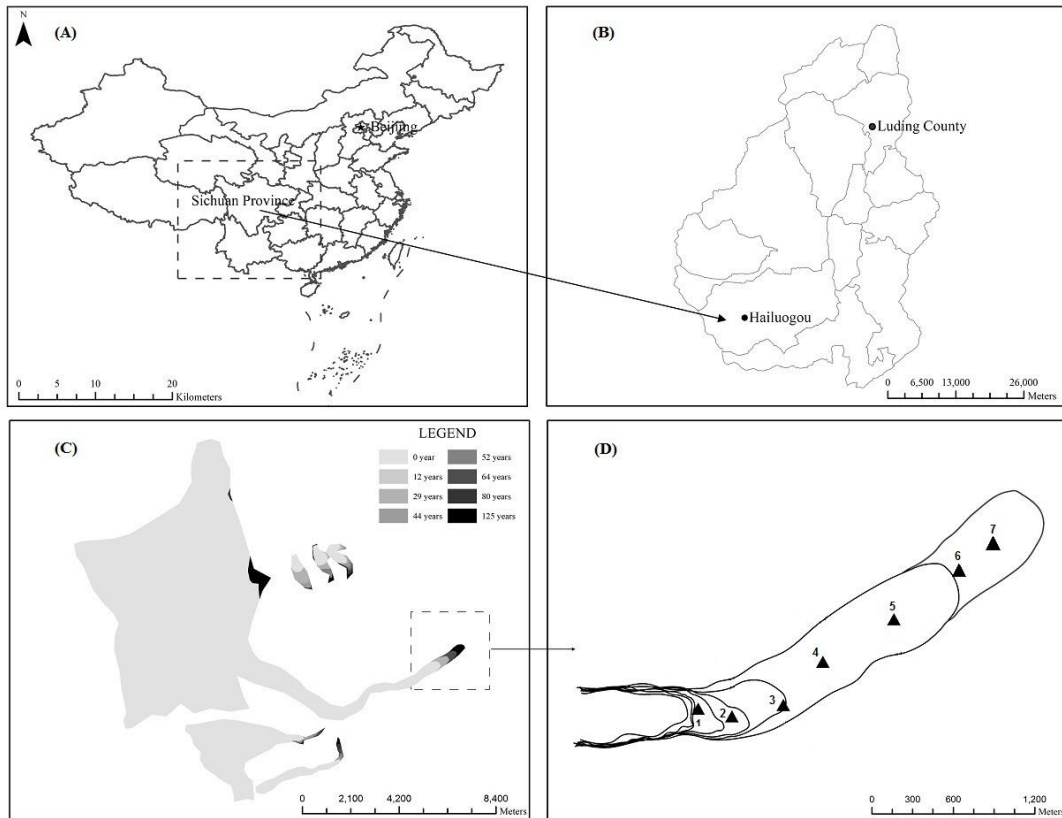

85

86

Supplement: FIG S1 [file mSystems.00475-20-sf001.pdf]
