# Supplementary material for: Plant and Soil Development Cooperatively Shaped the Composition of the phoD-Harboring Bacterial Community along the Primary Succession in the Hailuogou Glacier Chronosequence
Source: mSystems. 2020 Jul 28;5(4):e00475-20. doi: 10.1128/mSystems.00475-20 (PMC7394357; doi:10.1128/mSystems.00475-20)
Supplement: TABLE S4 [file mSystems.00475-20-st004.pdf]

	Rhizosphere			Bulk		
	R^2	*P*	Sig.	R^2	*P*	Sig.
pH	0.556	0.001	***	0.5289	0.001	***
Soil density	0.5665	0.001	***	0.4776	0.001	***
SOC	0.5562	0.001	***	0.662	0.001	***
TN	0.5887	0.001	***	0.6175	0.001	***
TP	0.0427	0.388		0.2227	0.011	*
AP	0.1535	0.038	*	0.1717	0.033	*
OP	0.4355	0.001	***	0.039	0.476	
TK	0.4574	0.001	***	0.5169	0.001	***
AK	0.0366	0.495		0.0088	0.843	
